# Labor in Moderately Contracted Pelves1Read before the Eighth District Branch of the Medical Society of the State of New York, Sept. 27, 1911.

**Published:** 1911-10

**Authors:** Francis C. Goldsborough

**Affiliations:** Professor of Obstetrics, University of Buffalo


### Labor in Moderately Contracted Pelves[1]

By DR. FRANCIS C. GOLDSBOROUGH,
Professor of Obstetrics, University of Buffalo

IT is my purpose, this afternoon, to discuss labor in moderately contracted pelves, and to attempt to bring out some of the points which will aid us in the treatment of these cases. I will not go into a detailed description of the various forms of pelvic deformity, but simply say a few words as to what is meant by a moderately contracted pelvis.

Generally speaking, any pelvis in which the diagonal conjugate is 11.50 cm. or less, is classed as contracted. However, we cannot base our treatment solely on the pelvic measurements, as the pelvis is only one factor, though a very important one, which must be considered in any given case. So, it will not be necessary to deal with figures, but simply to discuss the pelvis in general terms. By a moderate degree of contraction, we mean that large group of cases in which the pelvis varies from a trifle below normal to one in which we have a relative indication for Caesarean section,—*i.e.*, one having a true conjugate of 7 cm. or less.

When I say we will not discuss figures, I don't mean to imply that it is not of great importance to measure the pelvis. Careful pelvic measurements are of great assistance, for by them we can determine accurately the size of the pelvis; and in any given case, this knowledge is of the greatest importance.

In fact, if in all cases, the pelvis were measured some weeks before term, it would be of great value, for then the existence of any contraction would be recognised before an actual dystocia forced our attention to it.

The other factors, besides the pelvis, which will enter into the problem are:

The size and consistency of the foetal head;

The amount of molding it will undergo;

And the force of the uterine contractions.

Our ability to determine these factors before the onset of labor is, unfortunately, not very great. None of the methods of estimating the size of the head give us uniformly satisfactory results, and its consistency can only be approximately determined, after cervical dilatation enables us to palpate it and to determine the size of its sutures, and the degree of ossification of the bones. Practically, nothing can be told as to how the

1. Read before the Eighth District Branch of the Medical Society of the State of New York, Sept. 27, 1911.

head will mold until we see its behavior during the second stage of labor.

In primiparous women we have no way of judging before the onset of labor, of the force of the uterine contractions, because the general muscular development of the patient does not aid us. Multiparae, however, we can form some idea from the previous labors, though it must be remembered that often the contractions are apt to be less forcible with each succeeding labor.

Before considering treatment, it is well to remember that in the great majority of cases of moderately contracted pelves, the labor will end spontaneously, and the slighter the contraction, the higher the percentage will be. And even in quite marked contractions there will still be a goodly number of spontaneous deliveries.

From this it is evident that, whatever our method of treatment, it should include a thorough test of labor, whenever possible, before any operative procedure is determined upon.

It is impossible to give any fixed rules for operative interference, for each case must be studied as labor progresses, and a decision based on the conditions existing. In the majority of cases the child will present by the vertex, and cervical dilatation will be completed in a longer or shorter time,—when the membranes will rupture. Then, if after several hours of good pains, the head shows no signs of entering the pelvis, it is advisable to interfere, before the patient becomes exhausted, and the child is in bad condition.

However, when we have abnormal presentations, interference is often indicated as soon as the second stage is reached, because the position makes spontaneous labor impossible, and to allow the patient to continue in labor is useless and often harmful. In some cases, also, where the membranes rupture at the onset, or early in labor, interference is indicated before complete dilatation of the cervix.

It is well to remember that molding of the head occurs only after rupture of the membranes, and the number of hours the patient is in labor before the membranes rupture gives us no indication to interfere.

The methods of treatment which we will discuss are:
Induction of premature labor,
The use of the Walcher position,
Version and extraction,
Craniotomy,
Caesarean section,
Hebosteotomy (Pubiotomy.)

The induction of premature labor, from four to six weeks before term, is only rarely indicated in these cases, because of the high percentage of spontaneous labors that occur. And when premature labor is induced, the chances for the child are not very good, because of the difficulties attending the rearing of a premature infant. However, I feel that the induction of labor at term, or a few days before, has a distinct field. For, if the patient be allowed to go over time, we have a large child, with a firmly ossified head which does not mold readily, and often an unnecessarily difficult labor.

Before deciding upon any operation for delivery, it is always advisable to see what can be accomplished by the Walcher or hanging position. Often it will be found that with the patient in this position, under light anaesthesia for a short time, the head will enter the superior strait, when either spontaneous delivery will follow, or a comparatively safe and easy forceps can be done.

Formerly, not so much at the present time, podalic version and extraction was highly recommended in these cases. And it is true that the head will often come through the pelvis more easily by this means; but if version is to be done without serious injury to the mother, it must be performed early in the second stage of labor, and before the head has been given sufficient chance to mold and enter the pelvis. Another objection is that if there be any delay in delivering the after-coming head, the child will be lost.

Forceps, properly used, may be often indicated in these cases. But they should be used only after the head has become molded sufficiently to enter the superior strait. They should not be applied to the floating head and attempts made, with brute force, to drag a large head through a small pelvis.

In this group of cases, craniotomy is always indicated if the child dies during labor, or is dead when the patient is first seen. Here, of course, it is done in the interests of the mother, for it renders the delivery much easier and safer. Occasionally craniotomy on a living child is justifiable, when the surroundings of the patient are such that other operative procedures would subject the mother to too great risk. However, repeated craniotomies on the same woman are not justifiable, for the patient could be sent to a well-regulated maternity hospital, where the child could be safely delivered.

Caesarean section need rarely be considered, because we have seen how essential the test of labor is, and when this operation is done late in labor, the result is poor, so far as the mother is concerned.

In recent years, the operation of hebosteotomy (or, as it is commonly called, pubiotomy), has come into great favor; and it is especially indicated in this group of cases, for we can allow a thorough test of labor, trying the Walcher position, give time for molding of the head, and hope, if not for spontaneous labor, at least that the head will enter the pelvis, when forceps may be applied. Failing in this, we can still deliver the patient safely by means of hebosteotomy.

It must be remembered that with contracted pelves we are much more liable to find abnormal presentations, such as transverse and breech. And here the difficulties attending a decision of the best mode of treatment are even greater than with vertex presentations.

Here, however, there is a procedure which will aid us, if in the given case we fear that so much time will be required to deliver the after-coming head as to cause the death of the foetus. The patient can be prepared for pubiotomy, a Gigli saw passed behind the pubis, but before sawing through the bones, the delivery is proceeded with, and if, after a minute or two of traction on the after-coming head, it fails to enter the pelvis, the bone may be quickly sawed through, the delivery completed, and the child saved.

If, on the other hand, the child can be delivered without the necessity of severing the pubis, the skin incision may be closed, and no harm will have been done the patient.

The results of labor in moderately contracted pelves, for the mother should be but little different from labor in normal pelves, if the patient is properly handled, and even where pubiotomy is required the results are excellent. For the child, however, the matter is much more serious, and here the conduct of the labor, and especially the method of delivery will largely influence results. In a given case, the condition of the patient and foetus should be carefully watched; internal examination should be minimal and things should be left to nature as long as possible. However, the patient should not be allowed to continue in labor until exhaustion is present and the foetus is dying, before operative interference is begun.

The choice of operation will depend largely on the conditions present. If the head has entered the pelvis, the child can usually be delivered safely with forceps. If the head is still floating, pubiotomy will give very satisfactory results. While, in abnormal presentations and before attempting version and extraction, if the Gigli saw is put in place, we will still be able to save the child if difficulty is experienced in delivering the after-coming head.

